# Colonic Transendoscopic Enteral Tubing: Route for a Novel, Safe, and Convenient Delivery of Washed Microbiota Transplantation in Children

**DOI:** 10.1155/2021/6676962

**Published:** 2021-01-15

**Authors:** Min Zhong, Heena Buch, Quan Wen, Chuyan Long, Bota Cui, Faming Zhang

**Affiliations:** ^1^Medical Center for Digestive Diseases, The Second Affiliated Hospital of Nanjing Medical University, Nanjing 210011, China; ^2^Key Lab of Holistic Integrative Enterology, Nanjing Medical University, Nanjing 210011, China

## Abstract

**Aim:**

Colonic transendoscopic enteral tubing (TET) has been used for delivering fecal microbiota transplantation by washed preparation since 2015, which was recently named as washed microbiota transplantation (WMT). However, there are few reports available regarding the feasibility and safety of these studies in low-age population. This study is aimed at evaluating the safety, feasibility, and value of colonic TET in 3-7 years old children.

**Methods:**

All patients aged 3-7 years who underwent colonic TET in our center for WMT or medication were prospectively evaluated. The feasibility and safety of TET were evaluated. A questionnaire was completed by the children's parents to evaluate the children's response to the colonic TET as well as the parent's satisfaction.

**Results:**

Forty-seven children were included (mean age 5 years). TET was implemented into the colon of all the patients, and the success rate of the procedure was 100%. The median retention time of TET tube within the colon was 6 (IQR 5-7) days in 45 patients with tube falling out spontaneously, and the maximum retention time was up to 21 days. Multivariate analysis demonstrated that endoscopic clip number (*P* = 0.009) was an independent contributing factor for the retaining time of tube. With increase in the number of large clips, the retention time of TET tube was prolonged. No discomfort was reported during injection of the microbiota or medication suspension through the TET tube. During the follow-up, no severe adverse events were observed. All children's parents were satisfied with TET. Interestingly, the proportion of children's parents choosing TET as the delivery way of WMT increased from 29.79% before to 70.21% after TET (*P* < 0.001).

**Conclusions:**

This study, for the first time, demonstrates that colonic TET is a novel, safe, and convenient colonic delivery way for WMT and medication in children aged 3-7 years.

## 1. Introduction

The value of fecal microbiota transplantation (FMT) has grown exponentially in recent years. FMT has already been explored in the treatment of a variety of illnesses in children, other than recurrent *Clostridioides difficile* infection (CDI) [1], such as inflammatory bowel diseases (IBD) [2–4], allergic colitis [5], and gut-brain axis disease like autism [6] and epilepsy [7]. Along with studies on FMT in children, there are increasing number of studies highlighting the involvement of gut microbiota in various nongastrointestinal chronic disease like asthma, type 1 diabetes, Tourette's syndrome, etc. [8–10]. Similar to gut-brain-axis, another term called gut-skin-axis was recently termed for involvement of gut microbiota in skin disorders like atopic dermatitis [11, 12]. Although the evidence for FMT in children was mostly limited to case series and individual reports, FMT in pediatrics is important and promising.

The improved methodology of FMT based on the automatic washing process [13] and the related delivering consideration was coined as washed microbiota transplantation (WMT) by the consensus statement from the FMT-standardization Study Group in 2019 [14]. However, to deliver WMT in low-age children is more challenging than in adults, especially for those who are chronically ill and mentally immature, such as IBD and autism patients. There are three routes of delivering WMT, i.e., the upper gut, midgut, and lower gut [15, 16]; each method has its advantages and its limitations. Depending on the age, simple oral capsule administration is convenient for older children and adolescents but may not be feasible for young children [17]. Importantly, asphyxia may occur in children by oral capsules. WMT via colonoscopy is a typical choice, but patients cannot endure frequent bowel preparation and colonoscopy over a short period of time. Enema is an easy way of delivering fecal microbiota, but the access only arrives at the rectum and the sigmoid colon, making it difficult for children to hold the delivered microbiota for enough time. Therefore, in order to meet the needs of patients with multiple fresh WMTs or whole-colon administration of medications with one to two weeks, we developed a colonic delivery method for long-term maintenance of an indwelling, colonoscopically placed transanal enteral tube, which was called colonic transendoscopic enteral tubing (TET) [15, 18].

TET as a procedure has been reported as a safe and convenient procedure for multiple WMTs and colonic medication administration with a high degree of satisfaction among adult patients [15, 19–22]. The TET device (FMT Medical, Nanjing, China) was approved by National Medical Products Administration for endoscopic use since 2017. Allegretti et al. states that the TET is considered as a promising approach for FMT [23]. Recently, colonic TET has been recommended by the latest consensus from FMT-standardization Study group in Asia in 2019 [14] and an international FMT expert group in 2020 [24]. This method may be less psychologically challenging for patients than delivery of WMT via the upper and middle gut. The recent study reported that two to four large endoscopic clips could be recommended to maintain the TET tube within the colon for over 7 days in adults [18]. Our recent randomized controlled trial indicates that cap-assisted colonoscopy can reduce the time of second incubation of colonoscope in those colonoscopies with difficulty and decrease abdominal pain during endoscopy [25]. However, there were few data available regarding the feasibility and safety of these studies in low-age population. This study is aimed at evaluating the safety and feasibility of using colonic TET in pediatric patients aged 3-7 years, as well as evaluation of the possible affecting factors on the procedure. Furthermore, the perception and response of the children's parents related to the different delivery way of WMT have been assessed.

## 2. Patients and Methods

### 2.1. Patients

A prospective observational study was conducted at the Second Affiliated Hospital of Nanjing Medical University from May 2017 to January 2020. All patients met the inclusion criteria: age 3 to 7 years, suitability for endoscopy, and with parents' consent to undergo WMT and TET for children's diseases. Patients were excluded if they had severe intestinal stenosis, fistula, and risk of perforation during endoscopy; complication with serious anus lesions which might affect endoscopy; and no proper mucosa for endoscopic tissue clip fixation, the parents of the patients disagreed for the survey, or lost contacts. This study was approved by the Institutional Ethical Review Board of the Second Affiliated Hospital of Nanjing Medical University (2015KY042).

### 2.2. Colonic TET Procedure

**Figure 1:**
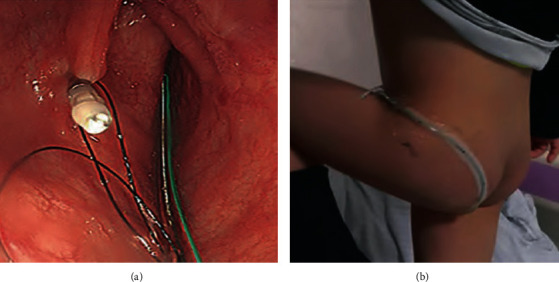
The procedure of colonic transendoscopic enteral tubing (TET). Under endoscopic guidance, the TET tube was fixed onto the mucosal fold of the colon with endoscopic clips (a). Nonrestricted leg movement of a 3-year-old child with a TET tube fixed onto the hip (b).

Regular colonoscopy, using a colonoscope with working channel diameter ≥ 3.2 mm, was performed under intravenous anesthesia. After complete evaluation of the colon, a soft TET tube (outer dimeter 2.7 mm, FMT medical, China) was inserted into the colon via the paraffin-lubricated colonoscope channel. Once the TET tube reached the target location (such as cecum), the colonoscope was carefully withdrawn, while keeping the tube in place. Then, the colonoscope was reinserted up to the target location, and the tube was fixed onto the wall with 1-4 endoscopic clips (ROOC-D-26-195-C, ≥10 mm, Nanjing Microtech Co.; HX-610-135 L, 135°, Olympus) along the three sites (named “the first site,” “the second site,” and “the third site,” each separated by 10 cm) on the distal part of the tube (Figure 1(a)). Generally, 1-2 clips at the first site and 0-2 clips at the second and/or the third site (as possibly required) were used. The location and number of the clips used for fixing the tube were chosen based on the mucosal folds, disease severity, and the duration for which the tube needs to be retained. The tube was secured with a medical tape on the right hip for easy access during the WMT administration (Figure 1(b)). The TET device was approved by China National Medical Products in 2017. The number, type, and location of the clips and procedure-related adverse events (AEs) were recorded for every patient. The TET tube retention time and method of tube expulsion were also recorded for statistical analysis.

### 2.3. WMT or Medication Delivery

Based on our previous reports on donor screening protocol for donors and automatic purification system (GenFMTer, FMT Medical, Nanjing, China) for microbiota from donated stool in a special lab [22] and the one-hour WMT protocol for WMT [7], the fecal microbiota suspension or medication suspension was delivered into the colon through TET tube. The right lateral position is recommended when delivering WMT or medication (such as mesalazine suspension). The microbiota (15-50 mL of suspension according to age in 1-2 min) or medication (e.g., mesalazine) solution should be injected at the temperature of 37°C. Patients are recommended to lay in 10° Trendelenburg position for 30 minutes after infusion and then in the supine position in order to prolong the retention of the infused fluid [19]. About 5 mL of saline is used to flush the tube after infusion. Retention of the microbiota suspension for over 1 hour indicates successful delivery of the microbiota through colonic TET.

### 2.4. Questionnaire

A questionnaire (Supplementary file available here), also approved by the Institutional Ethical Review Board of our hospital, was retrospectively given to the parents to evaluate their perspectives on colonic TET before and after the procedure, as well as to evaluate their children's responses to the colonic TET. The preferred delivery way of WMT before and after the procedure, parents' concerns prior to the procedure, parents' satisfaction, postprocedural change in motility/activity of the child, and child's toleration for TET were noted. Overall behavior of the patients was evaluated based on the parent's description. Among the five options offered, gastroscopy, colonoscopy, midgut TET, colonic TET, and enema, the parents were further asked which transplant route they preferred.

### 2.5. Clinical Evaluation of Colonic TET

The purpose, the success rate of the procedure, the fixation location, and the retaining time of the TET tube, as well as the type and number of endoscopic clips used were recorded. The retaining time is defined as the time from the implantation to natural shedding of the TET tube. Adverse events and the parents' satisfaction during and after TET were also recorded. Safety was evaluated in all patients by recording adverse events throughout long-term follow-up using the China microbiota transplantation system (http://www.fmtbank.org).

## 3. Statistical Analysis

The data were analyzed by using SPSS 21.0 (Chicago, IL, USA). Continuous variables were expressed using median and interquartile range. Categorical variables were summarized using absolute numbers and percentages. When the normality of the distribution of variables was acceptable, independent sample *t*-test was used. Comparisons of categorical variables between groups were performed using the chi-squared test. The relation between the retaining time and the endoscopic clips was evaluated using univariate and multivariable logistic regression analysis. A value of *P* < 0.05 (two-tailed) was considered significant.

## 4. Results

### 4.1. Characteristics of Patients

A total of 47 patients were included in this prospective study: 42 males and 5 females aged 3 to 7 years. As shown in Table 1, 45 (45/47, 95.74%) patients used TET for multiple WMTs and two (2/47, 4.26%) for WMT and intracolonic medication administration.

### 4.2. Feasibility of Colonic TET in Children

The colonic TET was successful performed in all 47 cases (100%). In 29 cases (61.70%), the tip (closed to mouth direction) of the TET tube was fixed in ileocecal region, transverse colon in 12 patients (25.53%), and the ascending colon in 6 patients (12.77%). Large clips were used on the sites of the TET tube in 35 cases during our preliminary observational period, 11 cases had one clip, 19 had two clips, three had three clips, and two had four clips. In the remaining 12 cases, small clips were used on the sites. In all cases, WMT or medication administration through colonic TET was successful. Two patients with UC were injected with mesalazine and steroids, respectively, through the TET tube after WMT until the TET tube fell off. After the treatment was completed, the TET tube naturally shed off in 45 patients (95.74%), and the median retaining time was 6 (IQR 5–7) days. The maximum retention time of the TET tube was up to 21 days.

### 4.3. Analysis on Retention Time of TET Tube

Of all the patients, 45 patients experienced natural expulsion of the TET tube. They were divided into the short-retaining time group (≤6 days) and the long-retaining time group (>6 days), considering 6 days as median retention time. As shown in Table 2, significant difference was observed between TET retaining time and the endoscopic clip number (*P* = 0.006) in the univariate analysis. Multivariate analysis demonstrated that only endoscopic clip number (*P* = 0.009) was an independent factor for affecting the retaining time. In patients with large endoscopic clips, we found that the number of endoscopic clips used significantly affected their retaining time (*P* = 0.006) (Table 3). In patients with small endoscopic clips, the retaining time of the TET tube significantly increased with the increased number of endoscopic clips (*P* = 0.025).

### 4.4. Optimal Methods of Performing WMT

**Figure 2:**
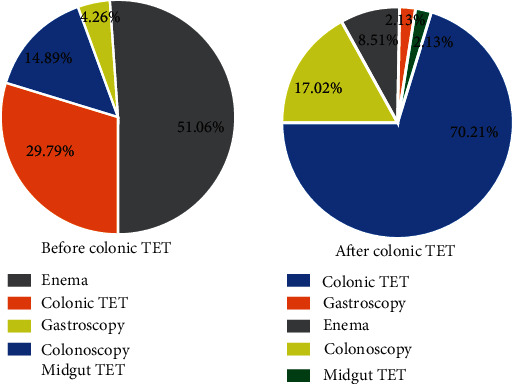
The most preferred delivery way of washed microbiota transplantation (WMT) by the children's parents before and after the procedure.

Among the five options offered for delivering WMT, the parents of the pediatric patients were asked which route of transplantation they would have preferred before and after the TET procedure. All the delivering ways were explained to the parents in detail, along with the pros and cons of each procedure. As shown in Figure 2, the most preferred choice for delivery of WMT before the procedure was enema (51.06%). This was obvious, given that enema is the least invasive procedure. Whereas after the colonic TET procedure, the colonic TET was the most preferred choice (70.21%) for the parents. The percentage of the first choice for colonic TET after the TET procedure was much higher than that before the TET procedure (29.79% vs. 70.21%, *P* < 0.001). Meanwhile, there were no parents who changed from the original acceptance attitude for colonic TET to not accepting it.

### 4.5. Safety and Satisfaction of the Colonic TET

During injection of the washed microbiota or medication suspension, through the TET, no mild to severe abdominal pain or diarrhea was reported. No severe AEs were observed during and after colonic TET. Among all patients with colonic TET, four parents (8.51%) complained that the tube affected their children's activities significantly during its retention period, and they (three of them were 3 years old) could not tolerate this change. This discomfort was largely due to the patients being too young, so we classified it as mild adverse events, definitely related to TET. All parents (100%) were satisfied with the colonic TET.

## 5. Discussion

WMT has shown a promising prospect for the treatment of dysbiosis-related diseases in children, but it is more challenging for them to undergo WMT or whole-colon medication. When compared to the adult population, repeated anesthesia, endoscopy, or enema, within short intervals, put children at greater risk; hence, we urgently need to explore a more convenient and safe delivery method. Colonic TET, as a new approach for colon-targeted drug delivery, was published for the first time in 2016 and has since been used in hospitals in China mainland [15, 19, 26–28] and China Taiwan since then [20]. In the present study, TET and WMT were successfully performed in all cases, and WMT or medicine retention time was longer than 1 hour. This indicates that colonic TET should be a feasible procedure in children.

In the present study, we found that the retaining time of colonic TET tube was significantly correlated with the number of endoscopic clips in children. Our results showed prolonged retention time of the TET tube with the increase of the number of large endoscopic clips. The retention time of the TET tube is related to the clinician's decision on the patient's condition. When multiple WMTs or a long-term intracolonic administration of medications is required, the TET tube should be retained for as long as possible, and it should be fixed with more endoscopic clips. However, the relationship between the type and number of endoscopic clips and the retention time should be evaluated in a larger sample size.

In previous studies, oral capsules or repeated endoscopic operation was the options for WMT in children [6, 29]. However, because of their young age and the psychological impact of long-term illness, it is difficult for children to cooperate with doctors to complete treatment. They cannot tolerate repeated invasive operation and swallow too many capsules. In a recent study about FMT-related adverse events, colonic TET was the route with the lowest incidence of delivery-related adverse events, at 6% [30]. In comparison, the incidence of delivery-related adverse events with FMT capsules was 29% [30]. Capsulized FMT has helped to overcome concerns of invasive administration but not other drawbacks [23], such as biting capsule, aspiration into trachea, and difficulty for taking too much. Thus, more research for capsulized FMT is required. Moreover, the effectiveness from a single WMT might be limited in severe and refractory microbiota-related conditions [22]. The colonic TET solves the limitations of the WMT input pathway to some extent [15]. It can not only complete multiple WMT treatments but also can be used for whole colonic administration of medication, avoiding intestinal injury and bleeding caused by repeated insertion of the enema tube or colonoscopy. And this is the only way which could be used for delivering medication while covering the whole colon, and there are no other methods which could be used for comparisons.

One of the major concerns of pediatricians about the use of TET techniques in children relates to their safety. Ding et al. reported that the FMT-related AEs associated with using colonic TET as the delivery method were lower when compared with the midgut [19]. Importantly, the latest systematic review showed that colonic TET was the pathway with the lowest incidence of delivery-related adverse events, compared with colonoscopy, enema, capsule, midgut tube, and gastroscopy [30]. In the present study, TET and WMT were successfully performed in all 47 cases (100%), and no severe TET-related complications occurred. The particles-caused tube obstruction was reported in another pilot study while delivering manually prepared fecal suspension [20]. However, there was no tube obstruction during WMT in the present study.

It should be emphasized that the TET tube does not affect the daily life of patients. Previous study in our center reported that 98.1% of adult patients were satisfied with WMT through TET [15]. In the present study, although some of the children's activities were restricted by TET, all parents were satisfied with TET. The preference for colonic TET became the first choice after the TET procedure, showing that the parents experienced no difficulty in handling their children with a colonic TET tube. Although there is no single best universal delivery method that matches all patients, the choice made should be patient-specific. When considering the delivery route of WMT in children, disease condition, aesthetic factors, psychology, convenience, and pain should be considered much more carefully than adults during the entire workflow [22]. Though bowel preparation may be slightly difficult among children, than adults, but is comparatively less of a mental burden than the existing chronic disease that affects their daily life.

To the best of our knowledge, this is the first study to survey the safety and feasibility of colonic TET in children. This study does, however, have some limitations. First, the sample size of this pilot study was too small for comparison of the retention time of colonic TET among different diseases, but a larger prospective study based on these preliminary results is ongoing. In addition, this study did not evaluate clinical responses to whole-colon administration compared with other traditional treatments that will be a part of our future studies.

## 6. Conclusions

In conclusion, this article, for the first time, reports the use of colonic TET tube in 3-7 years old children. The results demonstrate that the novel concept of colonic TET is a feasible, practical, and safe technique for multiple WMTs or frequent colonic medication administration, with a high degree of parents' satisfaction. The results highlight the significance of colonic TET as a technique for colon-targeted medication delivery in pediatric patients. The use of colonic TET is opening a new era of whole colonic administration with reducing the stress of physicians, patients, and their families.

## Figures and Tables

**Table 1:** Characteristics of 47 patients who underwent colonic TET.

Items	Results
Patients, *n*	47
Age, years, median (IQR)	5 (4–6)
Gender, male, *n* (%)	42 (89.36%)
Disease type, *n* (%)	
Autism	21 (44.68%)
Ulcerative colitis	6 (12.77%)
*Clostridioides difficile* infection	2 (4.26%)
Crohn's disease	1 (2.12%)
Others^∗^	17 (36.17%)
Disease duration, years, median (IQR)	2 (1–3.5)
Success rate of TET, %	100%
Location for fixing distal tube, *n* (%)	
Ileocecal	29 (61.70%)
Transverse colon	12 (25.53%)
Ascending colon	6 (12.77%)
Endoscopic clip type, *n* (%)	
Small endoscopic clip	12 (25.53%)
Large endoscopic clip	35 (74.47%)
Retaining time of TET tube, days, median (IQR)	6 (5-7)
Removal of tube, *n* (%)	
Naturally fell out	45 (95.74%)
Actively pulled out	2 (4.26%)
Satisfaction, %	100%
Purpose of TET, *n* (%)	
WMT	45 (95.74%)
WMT and medical administration	2 (4.26%)

WMT: washed microbiota transplantation; TET: transendoscopic enteral tubing. ^∗^Four cases with constipation, four with antibiotics-related dysbiosis, three with epilepsy, two with Tourette syndrome, two with atopic dermatitis, and two with allergic colitis.

**Table 2:** Univariate analysis for the retaining time of TET tube.

Items	Total	Short-retaining (≤6 days)	Long-retaining (>6 days)	*P* value
Patients, *n*	45	29	16	—
Gender, male, *n*	40	27	13	0.226
Age, years, mean ± SD	5.36 ± 1.25	5.59 ± 1.12	4.94 ± 1.39	0.196
Disease duration, years, median (IQR)	2 (1–3.5)	2 (1–4)	1.5 (1–3)	0.176
Fixed position	45	29	16	0.277
Ileocecal	28	18	10	
Nonileocecal	17	11	6	
Endoscopic clip type	45	29	16	0.222
Large endoscopic clip	33	23	10	
Small endoscopic clip	12	6	6	
Endoscopic clip number	2 (1.75-3)	2 (1–2)	3 (2–4)	0.006

SD: standard deviation; IQR: interquartile range.

**Table 3:** Correlation between the endoscopic clip number and TET retaining time.

	Endoscopic clip number	*N*	TET retaining time	*P* value
Small endoscopic clip	3	8	6 (5-7)	
	4	4	8 (5.5-15)	0.025
Large endoscopic clip	1	11	5 (4-6)	
	2	17	6 (6-7)	
	>2	5	8 (7-10)	0.006

## Data Availability

The questionnaire data used to support the findings of this study are included within the supplementary file.

## Supplementary Materials

Supplementary Materials A supplementary file is submitted separately, containing the questionnaire results.

## References

[1] Surawicz C. M., Brandt L. J., Binion D. G. (2013). Guidelines for diagnosis, treatment, and prevention of Clostridium difficile infections. *The American Journal of Gastroenterology*.

[2] Shimizu H., Arai K., Abe J. (2016). Repeated fecal microbiota transplantation in a child with ulcerative colitis. *Pediatrics International*.

[3] Pai N., Popov J. (2017). Protocol for a randomised, placebo-controlled pilot study for assessing feasibility and efficacy of faecal microbiota transplantation in a paediatric ulcerative colitis population: PediFETCh trial. *BMJ Open*.

[4] Goyal A., Yeh A., Bush B. R. (2018). Safety, clinical response, and microbiome findings following fecal microbiota transplant in children with inflammatory bowel disease. *Inflammatory Bowel Diseases*.

[5] Liu S. X., Li Y. H., Dai W. K. (2017). Fecal microbiota transplantation induces remission of infantile allergic colitis through gut microbiota re-establishment. *World Journal of Gastroenterology*.

[6] Kang D. W., Adams J. B., Gregory A. C. (2017). Microbiota Transfer Therapy alters gut ecosystem and improves gastrointestinal and autism symptoms: an open-label study. *Microbiome*.

[7] He Z., Cui B. T., Zhang T. (2017). Fecal microbiota transplantation cured epilepsy in a case with Crohn’s disease: the first report. *World Journal of Gastroenterol*.

[8] Bannier M., van Best N., Bervoets L. (2020). Gut microbiota in wheezing preschool children and the association with childhood asthma. *Allergy*.

[9] Leiva-Gea I., Sánchez-Alcoholado L., Martín-Tejedor B. (2018). Gut microbiota differs in composition and functionality between children with type 1 diabetes and MODY2 and healthy control subjects: a case-control study. *Diabetes Care*.

[10] Zhao H., Shi Y., Luo X., Peng L., Yang Y., Zou L. (2017). The effect of fecal microbiota transplantation on a child with Tourette syndrome. *Case reports in medicine*.

[11] Lee S. Y., Lee E., Park Y. M., Hong S. J. (2018). Microbiome in the gut-skin axis in atopic dermatitis. *Allergy, Asthma & Immunology Research*.

[12] Reddel S., del Chierico F., Quagliariello A. (2019). Gut microbiota profile in children affected by atopic dermatitis and evaluation of intestinal persistence of a probiotic mixture. *Scientific reports*.

[13] Zhang T., Lu G., Zhao Z. (2020). Washed microbiota transplantation vs. manual fecal microbiota transplantation: clinical findings, animal studies and in vitro screening. *Protein & Cell*.

[14] Fecal Microbiota Transplantation-standardization Study Group (2020). Nanjing consensus on methodology of washed microbiota transplantation. *Chinese Medical Journal*.

[15] Peng Z., Xiang J., He Z. (2016). Colonic transendoscopic enteral tubing: a novel way of transplanting fecal microbiota. *Endoscopy International Open*.

[16] Zhang F., Cui B., He X. (2018). Microbiota transplantation: concept, methodology and strategy for its modernization. *Protein & Cell*.

[17] Chen B., Avinashi V., Dobson S. (2017). Fecal microbiota transplantation for recurrent clostridium difficile infection in children. *The Journal of Infection*.

[18] Zhang T., Long C., Cui B. (2020). Colonic transendoscopic tube-delivered enteral therapy (with video): a prospective study. *BMC Gastroenterology*.

[19] Ding X., Li Q., Li P. (2019). Long-term safety and efficacy of fecal microbiota transplant in active ulcerative colitis. *Drug Safety*.

[20] Wang J. W., Wang Y. K., Zhang F. (2019). Initial experience of fecal microbiota transplantation in gastrointestinal disease: a case series. *The Kaohsiung Journal of Medical Sciences*.

[21] Ding X., Li Q., Li P. (2020). Fecal microbiota transplantation: a promising treatment for radiation enteritis?. *Radiotherapy and oncology: journal of the European Society for Therapeutic Radiology and Oncology*.

[22] Zhang F., Zhang T., Zhu H., Borody T. J. (2019). Evolution of fecal microbiota transplantation in methodology and ethical issues. *Current opinion in pharmacology*.

[23] Allegretti J. R., Mullish B. H., Kelly C., Fischer M. (2019). The evolution of the use of faecal microbiota transplantation and emerging therapeutic indications. *Lancet*.

[24] Ianiro G., Mullish B. H., Kelly C. R. (2020). Reorganisation of faecal microbiota transplant services during the COVID-19 pandemic. *Gut*.

[25] Wen Q., Liu K. J., Cui B. T. (2020). Impact of cap-assisted colonoscopy during transendoscopic enteral tubing: a randomized controlled trial. *World Journal of Gastroenterology*.

[26] Xie W. R., Yang X. Y., Xia H. H., Wu L. H., He X. X. (2019). Hair regrowth following fecal microbiota transplantation in an elderly patient with alopecia areata: a case report and review of the literature. *World Journal of Clinical Cases*.

[27] Huang H. L., Chen H. T., Luo Q. L. (2019). Relief of irritable bowel syndrome by fecal microbiota transplantation is associated with changes in diversity and composition of the gut microbiota. *Journal of Digestive Diseases*.

[28] Ye Z. N., Xia H. H. X., Zhang R. (2020). The efficacy of washed microbiota transplantation on Helicobacter pylori eradication: a pilot study. *Gastroenterology Research and Practice*.

[29] Nicholson M. R., Mitchell P. D., Alexander E. (2019). Efficacy of fecal microbiota transplantation for Clostridium difficile infection in children. *Clinical Gastroenterology and Hepatology*.

[30] Marcella C., Cui B., Kelly C. R., Ianiro G., Cammarota G., Zhang F. (2021). Systematic review: the global incidence of faecal microbiota transplantation-related adverse events from 2000 to 2020. *Alimentary Pharmacology & Therapeutics*.

