# Supplementary material for: Colonic Transendoscopic Enteral Tubing: Route for a Novel, Safe, and Convenient Delivery of Washed Microbiota Transplantation in Children
Source: Gastroenterol Res Pract. 2021 Jan 15;2021:6676962. doi: 10.1155/2021/6676962 (PMC7826206; doi:10.1155/2021/6676962)
Supplement: Supplementary Materials — A supplementary file is submitted separately, containing the questionnaire results. [file 6676962.f1.docx]

**Questionnaire Results**

1. What was your first choice for the way of washed microbiota transplantation (WMT) administration before the procedure? (Based on the co-operation of your child) please answer just according to your thinking before your child underwent colonic TET.

| **First choice** | **No.** | **Percentage** |
| --- | --- | --- |
| A. Gastroscopy (Single WMT) | 2 | 4.26% |
| B. Colonoscopy (Single WMT) | 7 | 14.89% |
| C. Midgut TET (Multiple WMT) | 0 | 0% |
| D. Colonic TET (Multiple WMT) | 14 | 29.79% |
| E. Enema (Multiple WMT) | 24 | 51.06% |
| Total | 47 | 100% |

1. How does colonic TET affect the mobility/ activity of your child?

| **Options** | **No.** | **Percentage** |
| --- | --- | --- |
| A. No change in Activity | 23 | 48.94% |
| B. Mild- moderate change in activity but acceptable | 20 | 42.55% |
| C. Significantly low motility | 4 | 8.51% |
| Total | 47 | 100% |

1. How does your child tolerate colonic TET?

| **Options** | **No.** | **Percentage** |
| --- | --- | --- |
| A. Uncomfortable and cannot tolerate | 2 | 4.26% |
| B. Mild discomfort but can tolerate | 40 | 85.11% |
| C. No special feeling | 5 | 10.64% |
| Total | 47 | 100% |

1. Are you satisfied with the use of colonic TET for WMT?

| **Options** | **No.** | **Percentage** |
| --- | --- | --- |
| A. Satisfactory | 47 | 100% |
| B. Not Satisfactory | 0 | 0% |
| Total | 47 | 100% |

1. What were your concerns prior to the procedure?

| **Options** | **No.** | **Percentage** |
| --- | --- | --- |
| A. Use of Anesthesia | 26 | 55.32% |
| B. Whether my child will be able to pass stool normally or not | 1 | 2.13% |
| C. Side effects/ Adverse events (abdominal pain/ abdominal discomfort/ anal pain) | 20 | 42.55% |
| Total | 47 | 100% |

1. What was your first choice for the way of washed microbiota transplantation (WMT) administration after the procedure? (Based on the co-operation of your child) please answer just according to your thinking after your child experienced colonic TET.

| **Options** | **No.** | **Percentage** |
| --- | --- | --- |
| A. Gastroscopy (Single WMT) | 1 | 2.13% |
| B. Colonoscopy (Single WMT) | 8 | 17.02% |
| C. Midgut TET (Multiple WMT) | 1 | 2.13% |
| D. Colonic TET (Multiple WMT) | 33 | 70.21% |
| E. Enema (Multiple WMT) | 4 | 8.51% |
| Total | 47 | 100% |
